# 

**Published:** 1918

**Authors:** 


					_  SPRING NUMBER, 1918.
Vol. XXXVI. No. 135.
JUL 39 (gig
THE
BRISTOL
MEDICO-CHIRURGICAL
PCRUSHEl) UiVDKlt TUB AUSt'ICES OF
THE BRISTOL MEDICO-CH1RURGICtf&SOCI
\ J!jNl3
Vk ?
Editor: P. WATSON-WILLIAMS,>M.D.,
WITH WHOM ARE ASSOCIATED v -? ?
J. LACY FIRTH, M.S., M.D., JAMES SWAIN. C.B., M.S., M.I).
J. A. NIXON, B.A., M.D., Assistant Editor.
Acting Editorial Secretary: W. A. SMITH, M.A., M.B.
BRISTOL: J. W. ARROWSMITH LTD.
London : Simpkin, Marshall, Hamilton, Kent & Co. Ltd.
Price One Shilling and Sixpence net.

				

